# Supplementary material for: Design and Evaluation of Chimeric Plasmodium falciparum Circumsporozoite Protein-Based Malaria Vaccines
Source: Vaccines (Basel). 2024 Mar 25;12(4):351. doi: 10.3390/vaccines12040351 (PMC11053680; doi:10.3390/vaccines12040351)
Supplement: Supplementary file 1 [file vaccines-12-00351-s001.zip › vaccines-2905742-supplementary.pdf]

Supplemental Material

Design and Evaluation of Chimeric *Plasmodium falciparum* Circumsporozoite

Protein-Based Malaria Vaccines

William H. Stump [†], Hayley J. Klingenberg [†], Amy C. Ott, Donna M. Gonzales and James M. Burns, Jr. *

Center for Molecular Parasitology, Department of Microbiology and Immunology, Drexel University College of Medicine, 2900 Queen Lane, Philadelphia, PA 19129, USA

*Correspondence: jmb53@drexel.edu

[†] These authors contributed equally to this work.

Table S1
15-mer overlapping peptides of r*Pf*CSP(#1)

Peptide Number	Peptide Sequence	Peptide Location
1	MAHHHHHHPGGSGSG	1-15
2	HHHPGGSGSGTSGSS	6-20
3	GSGTSGSSSNTRVLN	13-27
4	SSNTRVLNELNYDNA	20-34
5	NELNYDNAGTNLYNE	27-41
6	AGTNLYNELEMNYYG	34-48
7	ELEMNYYGKQENWYS	41-55
8	GKQENWYSLKKNSRS	48-62
9	SLKKNSRSLGENDDG	55-69
10	SLGENDDGNNNNGDN	62-76
11	GNNNNGDNGREGKDE	69-83
12	NGREGKDEDKRDGNN	76-90
13	EDKRDGNNEDNEKLR	83-97
14	NEDNEKLRKPKHKKL	90-104
15	RKPKHKKLKQPGDGN	97-111
16	LKQPGDGNPDPNANP	104-118
17	NPDPNANPNVDPNAN	111-125
18	PNVDPNANPNVDPNA	118-132
19	NPNVDPNANPNVDPN	125-139
20	ANPNVDPNANPNANP	132-146
21	PNANPLKNANPNANP	146-160
22	NANPNANPNANPNAN	Central Repeat
23	NANPNANPNANPNKN	181-195
24	PNANPNKNNQGNGQG	188-202
25	NNQGNGQGHNMPNDP	195-209
26	GHNMPNDPNRNVDEN	202-216
27	PNRNVDENANANNAV	209-223
28	NANANNAVKNNNNEE	216-230
29	VKNNNNEEPSDKHIE	223-237
30	EPSDKHIEQYLKKIK	230-244
31*	EQYLKKIKNSISTEW	237-251
32	KNSISTEWSPCSVTC	244-258
33*	WSPCSVTCGNGIQVR	251-265
34*	CGNGIQVRIKPGSAN	258-272
35*	RIKPGSANKPKDELD	265-279
36*	NKPKDELDYENDIEK	272-286
37*	DYENDIEKKICKMEKCSS	279-296

*** Peptide Pool 6 for T cell assays**

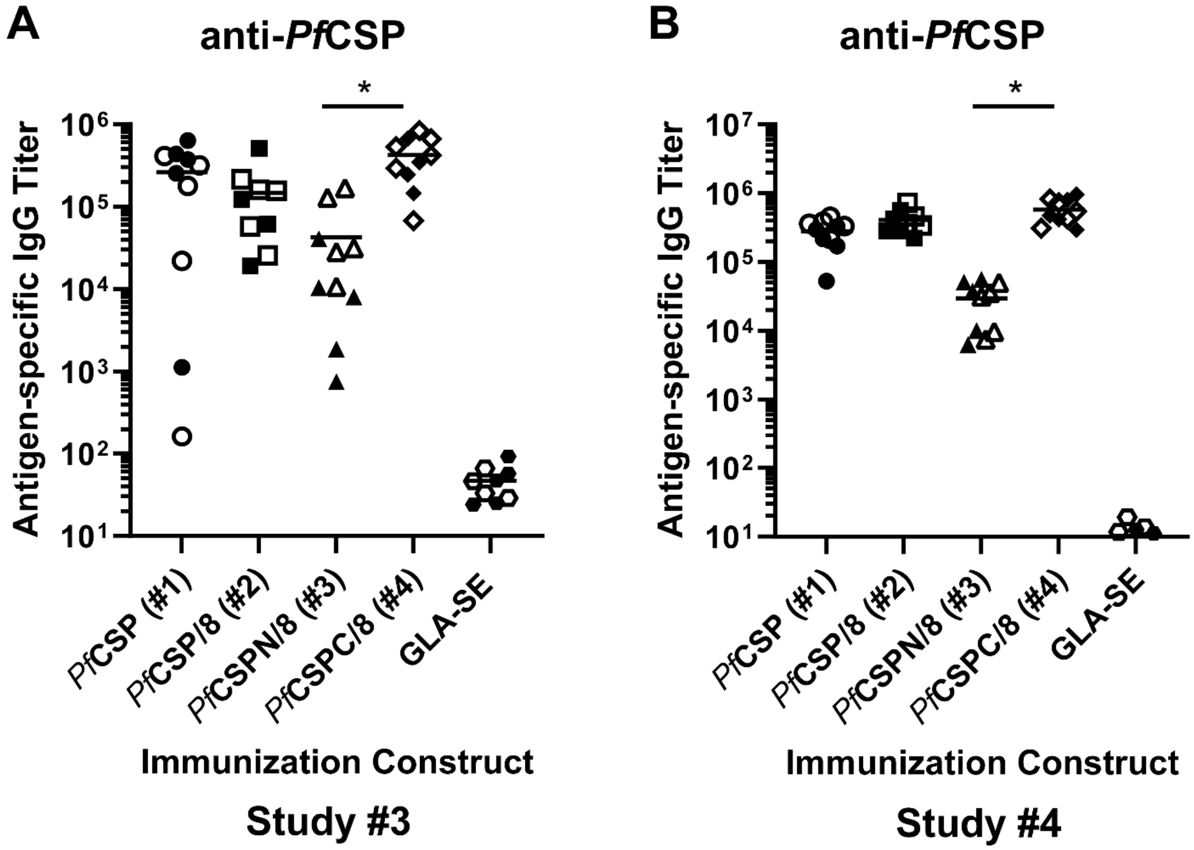

Figure S1. Magnitude of vaccine-induced antibody responses in immunization studies 3 and 4. Antigen-specific IgG titers were determined by ELISA on wells coated with *Pf*CSP (#1) using secondary sera from CD1 mice (n=10 per group; female, open symbols; male, closed symbols) immunized with r*Pf*CSP (#1, circles), r*Pf*CSP/8 (#2, squares), r*Pf*CSPN/8 (#3, triangles), r*Pf*CSPC/8 (#4, diamonds) or GLA-SE (hexagons) in study #3 (panel A) and study #4 (panel B). Statistical significance of differences in titers were assessed by the Kruskal-Wallis test followed by Dunn's correction for multiple comparisons. * $p < 0.05$.

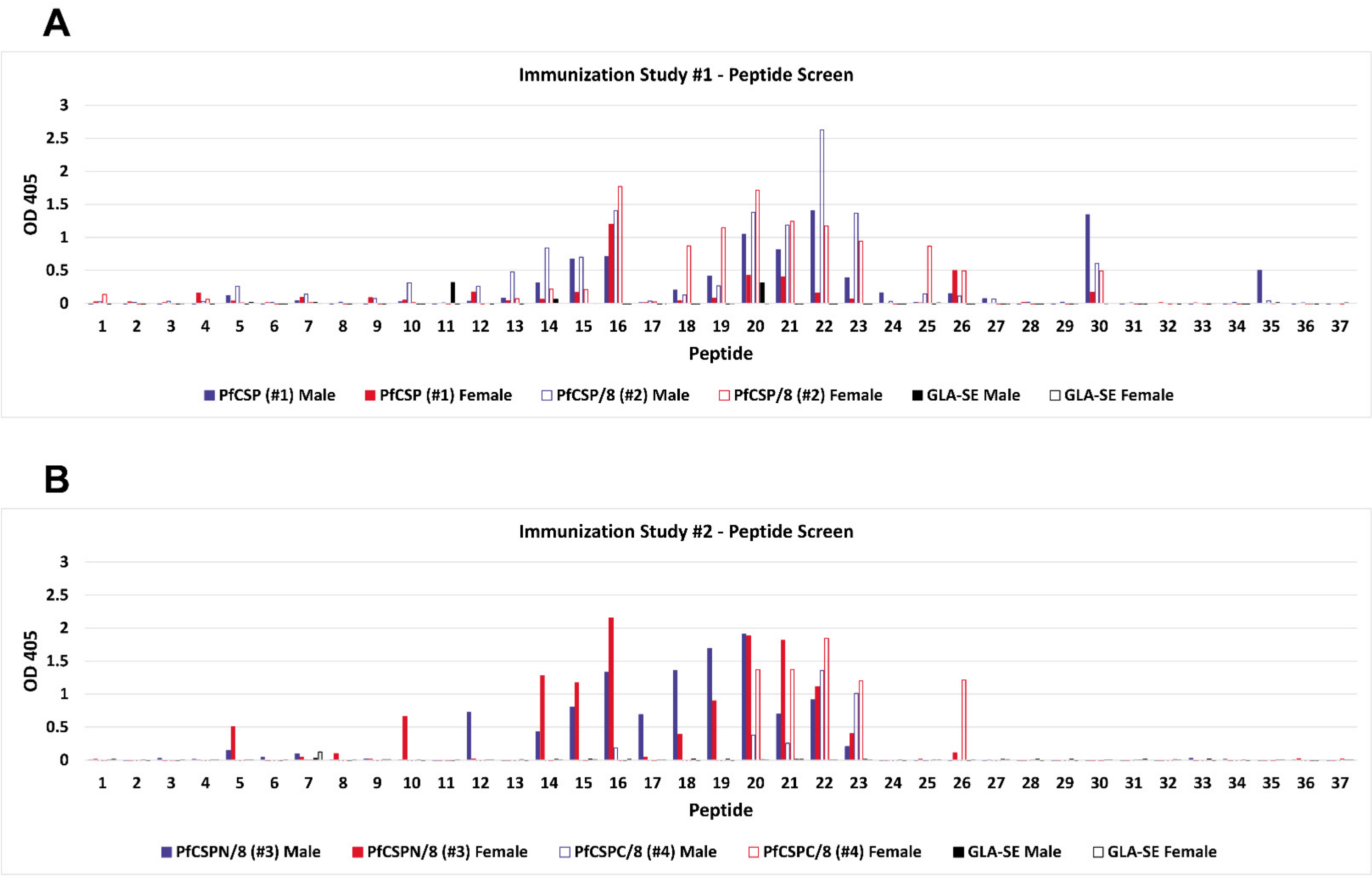

Figure S2. Initial screen of the specificity of r*Pf*CSP vaccine-induced antibodies. Pools of sera from male (n=5, blue) and female (n=5, red) mice immunized with r*Pf*CSP (#1), r*Pf*CSP/8 (#2), r*Pf*CSPN/8 (#3), or r*Pf*CSPC/8 (#4) were assessed by ELISA for reactivity with 37 overlapping 15 amino acid, biotinylated peptides spanning the length of *Pf*CSP (#1). Sera from Study #1 is shown in **(A)** and Study #2 is shown in **(B)**. The OD_{405} using sera assayed at a dilution of 1:1000 is plotted for each peptide in the array.

A – Figure 2A, main manuscript

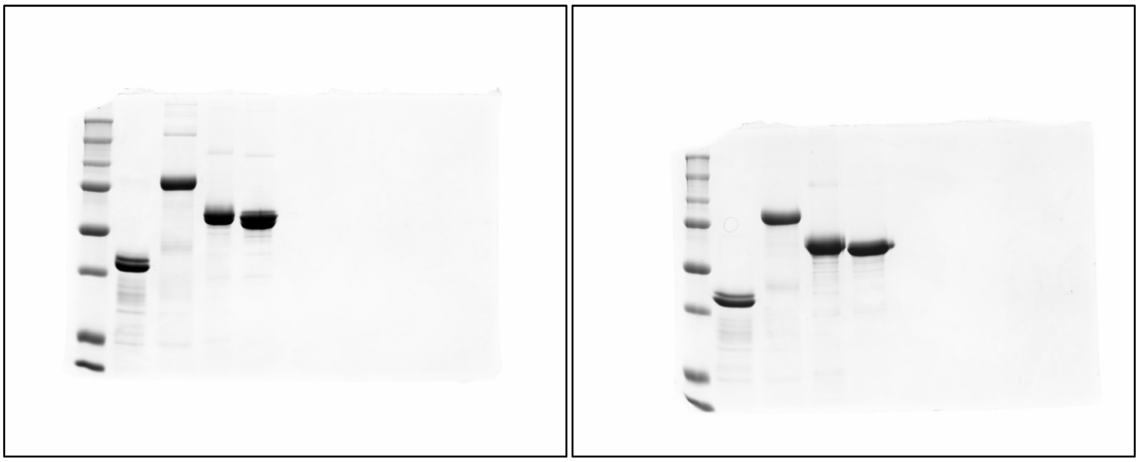

Non-reduced **Reduced**

B – Figure 2B, main manuscript[t

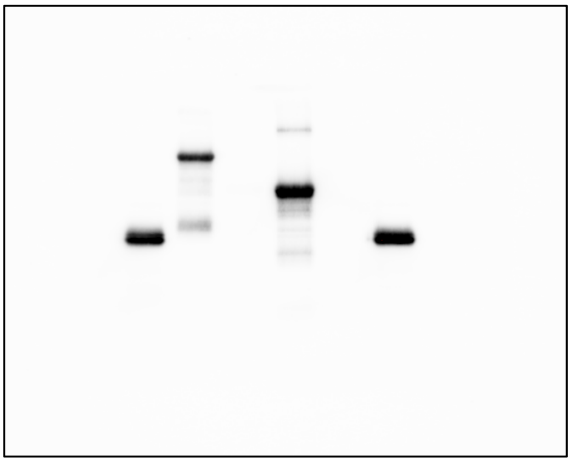

Figure S3. Assessment of purified r*Pf*CSP-based vaccines, original images. **(A)** Full images of Figure 2A – Coomassie-blue stained SDS-PAGE gel of purified r*Pf*CSP-based vaccines (#1-#4) run under non-reducing (left panel) and reducing (right panel) conditions. **(B)** Full image of Figure 2B immunoblot of purified, non-reduced r*Pf*CSP-based vaccines (#1-#4) using mAb 4B3.
